# Deep learning enables accurate soft tissue tendon deformation estimation in vivo via ultrasound imaging

**DOI:** 10.1038/s41598-024-68875-w

**Published:** 2024-08-08

**Authors:** Reece D. Huff, Frederick Houghton, Conner C. Earl, Elnaz Ghajar-Rahimi, Ishan Dogra, Denny Yu, Carisa Harris-Adamson, Craig J. Goergen, Grace D. O’Connell

**Affiliations:** 1 Department of Mechanical Engineering, University of California, Berkeley, Berkeley, CA 94720 USA; 2 Weldon School of Biomedical Engineering, Purdue University, West Lafayette, IN 47907 USA; 3 School of Industrial Engineering, Purdue University, West Lafayette, IN 47906 USA; 4 School of Public Health, University of California, Berkeley, Berkeley, CA 94704 USA; 5 Department of Occupational and Environmental Medicine, University of California, San Francisco, San Francisco, CA 94117 USA; 6 Department of Orthopaedic Surgery, University of California, San Francisco, San Francisco, CA 94142 USA

**Keywords:** Deep learning, StrainNet, Image texture correlation, Biomechanics, Biomedical engineering, Mechanical engineering

## Abstract

Image-based deformation estimation is an important tool used in a variety of engineering problems, including crack propagation, fracture, and fatigue failure. These tools have been important in biomechanics research where measuring in vitro and in vivo tissue deformations are important for evaluating tissue health and disease progression. However, accurately measuring tissue deformation in vivo is particularly challenging due to limited image signal-to-noise ratio. Therefore, we created a novel deep-learning approach for measuring deformation from a sequence of images collected in vivo called StrainNet. Utilizing a training dataset that incorporates image artifacts, StrainNet was designed to maximize performance in challenging, in vivo settings. Artificially generated image sequences of human flexor tendons undergoing known deformations were used to compare benchmark StrainNet against two conventional image-based strain measurement techniques. StrainNet outperformed the traditional techniques by nearly 90%. High-frequency ultrasound imaging was then used to acquire images of the flexor tendons engaged during contraction. Only StrainNet was able to track tissue deformations under the in vivo test conditions. Findings revealed strong correlations between tendon deformation and applied forces, highlighting the potential for StrainNet to be a valuable tool for assessing rehabilitation strategies or disease progression. Additionally, by using real-world data to train our model, StrainNet was able to generalize and reveal important relationships between the effort exerted by the participant and tendon mechanics. Overall, StrainNet demonstrated the effectiveness of using deep learning for image-based strain analysis in vivo.

## Introduction

Image-based deformation measurement has been utilized in many engineering problems, such as crack propagation^1^, fracture^2^, and fatigue^3^. When applied to medical images, these techniques have aided in the disease diagnostics^4–7^, assessment of injury mechanisms^8–11^, and evaluation of disease pathology^6,12–14^. Interest in using non-invasive approaches for tracking deformation in vivo has grown, due to its potential in assessing rehabilitation strategies or disease progression^15,16^.

Specifically, non-invasive approaches for measuring in vivo tissue deformation have relied on magnetic resonance (MR) or ultrasound imaging. MR imaging provides higher resolution images of soft tissues^17^; however, MR scans are costly and require multiple minutes to acquire a single image, which is not ideal for imaging dynamic loading. In contrast, ultrasound imaging provides faster, low-cost images, at the trade-off of image resolution. Therefore, there is significant interest developing methods to accurately measure tissue strains with ultrasound images. For example, high-precision strain mapping has been instrumental for identifying early stages of tendon disorders and monitoring their progression, thereby informing preventative and therapeutic strategies^4–7^. In clinical settings, such imaging may be particularly valuable for managing tendinopathy, a condition common among athletes and older adults^18^. Non-invasive strain measurements may also enable practitioners to evaluate rehabilitation protocol effectiveness and forecast patient prognoses^19,20^. Nonetheless, translation of tissue strain measurement techniques to routine clinical practice is hindered by technical difficulties associated with capturing images that are not affected by patient movement, noise, or resolution^21–34^. Therefore, it is important to develop strain measurement techniques that can perform well under clinical settings^9,35^.

Various techniques have been employed to calculate tissue deformations, which is essential for understanding tendon mechanics. Techniques such as digital image correlation (DIC)^36^ and direct deformation estimation (DDE)^22^ are prevalent for quantifying tendon deformation. These methods have been applied in various in vivo studies^37–40^. Nonetheless, they are challenged in the presence of low signal-to-noise environments and can suffer from limited spatial resolution^22–34^ (Section S1 in the Supplementary Information). Specifically, DIC, DDE, and other texture-based image correlation techniques may encounter difficulties in situations with poor contrast or images with insufficient intensity gradients. Consequently, researchers are often constrained to point-wise strain measurements within the tendon, that limits the spatial resolution of strain mapping, particularly during dynamic tendon loading^41–44^.

More recently, developments in machine learning have shown promise in measuring strains and this approach may present benefits over traditional strain measurement methods^45–48^. In particular, deep learning techniques, such as convolutional neural networks (CNNs), have been applied to predict strain maps between successive images. These methods resulted in more accurate and robust measurements compared to traditional image texture correlation techniques, such as DIC, in controlled in vitro settings^46^. The training required for deep learning approaches also provides an advantage over traditional methods, because training the model with a large dataset of known strains allows the model to ignore image artifacts that can reduce accuracy^45–48^. Thus, the technique can be applied to a wider range of images, including those with lower signal-to-noise ratios, which can be challenging for traditional image texture correlation methods (Section S1 in the Supplementary Information). Overall, the use of deep learning for image-based strain measurement has the potential to greatly improve assessment and understanding of in vivo tissue mechanics, particularly in the context of soft tissue under dynamic loading conditions.

Here, we propose a deep-learning approach, called StrainNet, specifically designed to maximize performance in challenging, in vivo settings. StrainNet utilizes a two-stage CNN architecture to predict full-field strain maps from a sequence of images that may be acquired in a medical setting (e.g., ultrasound images). The network was trained using a customized dataset, based on observations of tissue deformation and image artifacts in vivo, allowing it to overcome image artifacts that hamper traditional methods, such as image noise and artifacts, and provide accurate, full-field deformation predictions. We test and validate StrainNet on synthetic images with known deformations and real, experimentally collected ultrasound images of the flexor tendon in tension. Our results demonstrate that StrainNet outperforms traditional image texture correlation algorithms in both synthetic and real in vivo datasets, ultimately revealing strong correlations between tendon strains and applied forces as well as between load magnitude and measured mechanical properties in vivo. It is important to note that while StrainNet can provide more accurate information in these challenging situations, it requires significant training to achieve such accuracy. The design and capabilities of StrainNet hold immense potential for tracking deformation during both loading and unloading phases, leading to substantial progress in assessing soft tissue deformation. Our models and a tutorial for utilizing StrainNet are freely available at strainnet.github.io.

## Results

### Human flexor tendons undergoing contraction

**Figure 1:**
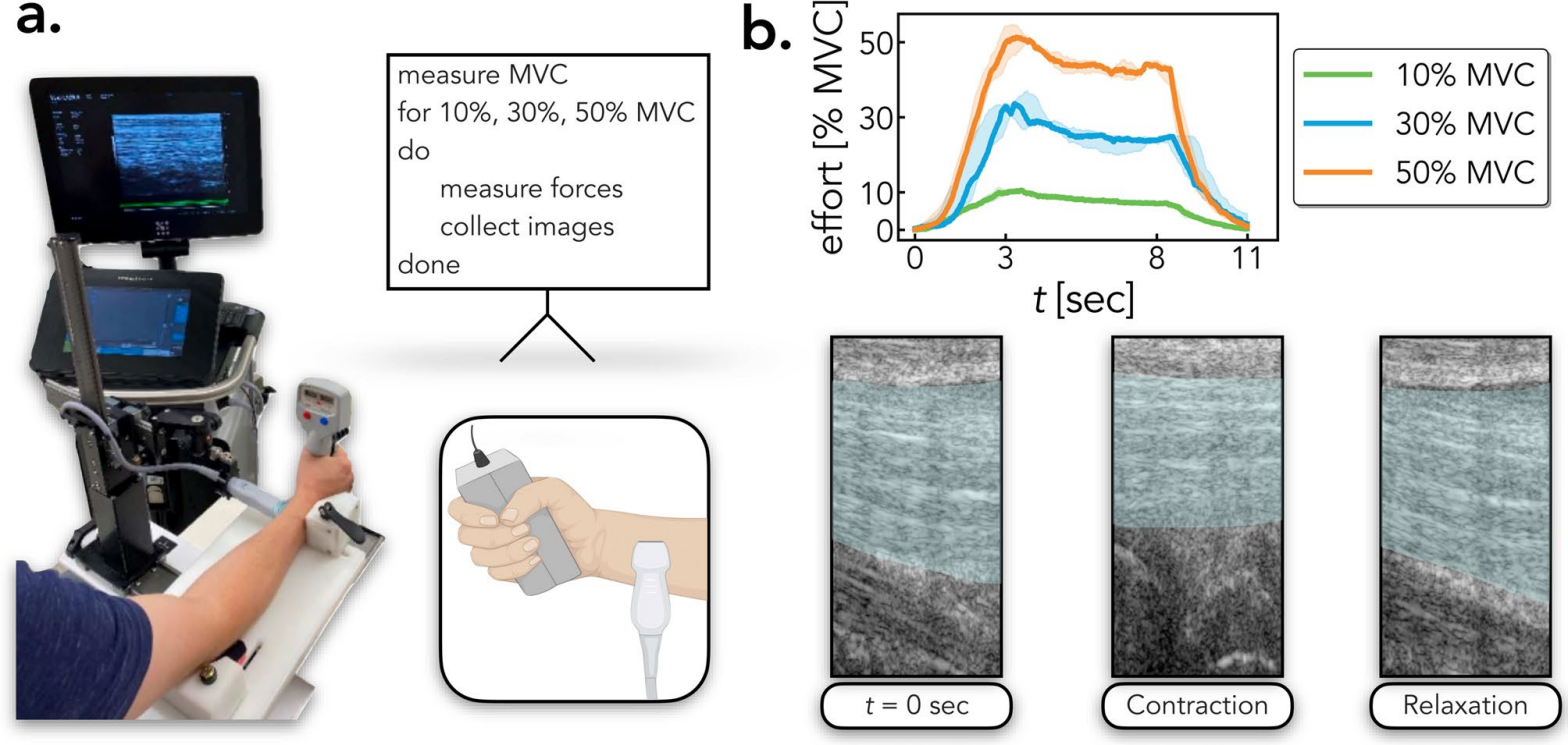
Experimental protocol, measured forces, and ultrasound images. (**a**) Custom mount with the participant gripping the dynamometer to measure forces while high-frequency ultrasound images were collected. (**b**) Once the participant’s maximum voluntary contraction (MVC) was determined, the participant was asked to squeeze the dynamometer to 10%, 30%, and 50% MVC. Data is shown throughout the contraction, hold, and relaxation for 10%, 30%, and 50% MVC. Characteristic images of the *flexor digitorum superficialis* (FDS) tendon, represented in teal, during the initiation of the test, during contraction (i.e., hold period), and after relaxation.

To evaluate StrainNet’s ability to predict tissue deformation, we designed a custom testing jig that allowed a participant to squeeze a dynamometer, providing grip forces, while simultaneously collecting in vivo images of their flexor digitorum superficialis (FDS) tendon with an ultrasound probe (Fig. 1a; MicroFET, Hoggan Scientific, Salt Lake City, UT, United States). The custom-built testing jig reduced movement of the forearm, and reduced out-of-plane movement of the FDS during testing and imaging (Fig. 1a). A high-frequency ultrasound probe was used to continuously collect images along the long axis of the FDS throughout the test protocol (Vevo3100 Ultrasound Imaging System, FUJIFILM VisualSonics Inc., Toronto, Ontario Canada; 21 MHz center frequency linear array ultrasound transducer; 15-30 MHz bandwidth; MX250). A participant was then asked to grip the dynamometer to their maximal effort to determine their maximum voluntary contractions (MVC). Averaged over the three trials, the participant’s MVC was 289.8N. The participant was then asked to contract their forearm to three different effort levels—10%, 30%, and 50% of their MVC—in 3 s, hold the contraction for 5 s, and relax in 3 s (Fig. 1a). Each effort level was repeated five times for a total of fifteen trials (*n* = 15); however, data from two trials were lost due to corruption of the data file. Informed consent was obtained from all participants, and all of the trials were performed with Purdue Institutional Review Board approval (IRB-2020-497) and in accordance with the Declaration of Helsinki.

The participant maintained the target MVC throughout the 5 s hold period with only a maximum 16% difference between the desired and measured MVC (Fig. 1b; Section S2 in the Supplementary Information). Over the course of the loading period, the tendon elongated and translated upward before returning to its original position after relaxation (Fig. 1b). The testing configuration enabled robust evaluation of StrainNet’s performance in predicting tissue deformation under a range of physiological strains, including those that may be encountered during daily activities.

### StrainNet outperforms traditional techniques in controlled environments

To test the accuracy of our strain analysis method in a controlled environment, five synthetic test cases were created by artificially imposing a non-linear strain field onto ultrasound images of the FDS tendon. The test cases simulated the experimental procedure with contraction, hold, and relaxation periods, as described above. The prescribed non-linear strain field was designed to reflect reported observations of in vivo tendon mechanics. Specifically, the strain in the superficial layer of the tendon was set to 75% of the deep layer^49^, and the tendon was modeled as an incompressible material^40,50^. The five test cases differed in their maximum longitudinal strain, $\epsilon _{long}^{max}$, which was set to 4%, 7%, 10%, 13%, and 16% to cover the range of reported in vivo strains^38–40,49^. Noise representative of that present in ultrasound imaging was added to all synthetic test cases to emulate challenges present in the experimental dataset. A complete description of the synthetic test cases is provided in the Supplemental Information S5. By using synthetic test cases with known deformations, we were able to compare the performance and accuracy of our deep learning based approach with existing texture correlation algorithms.

**Figure 2:**
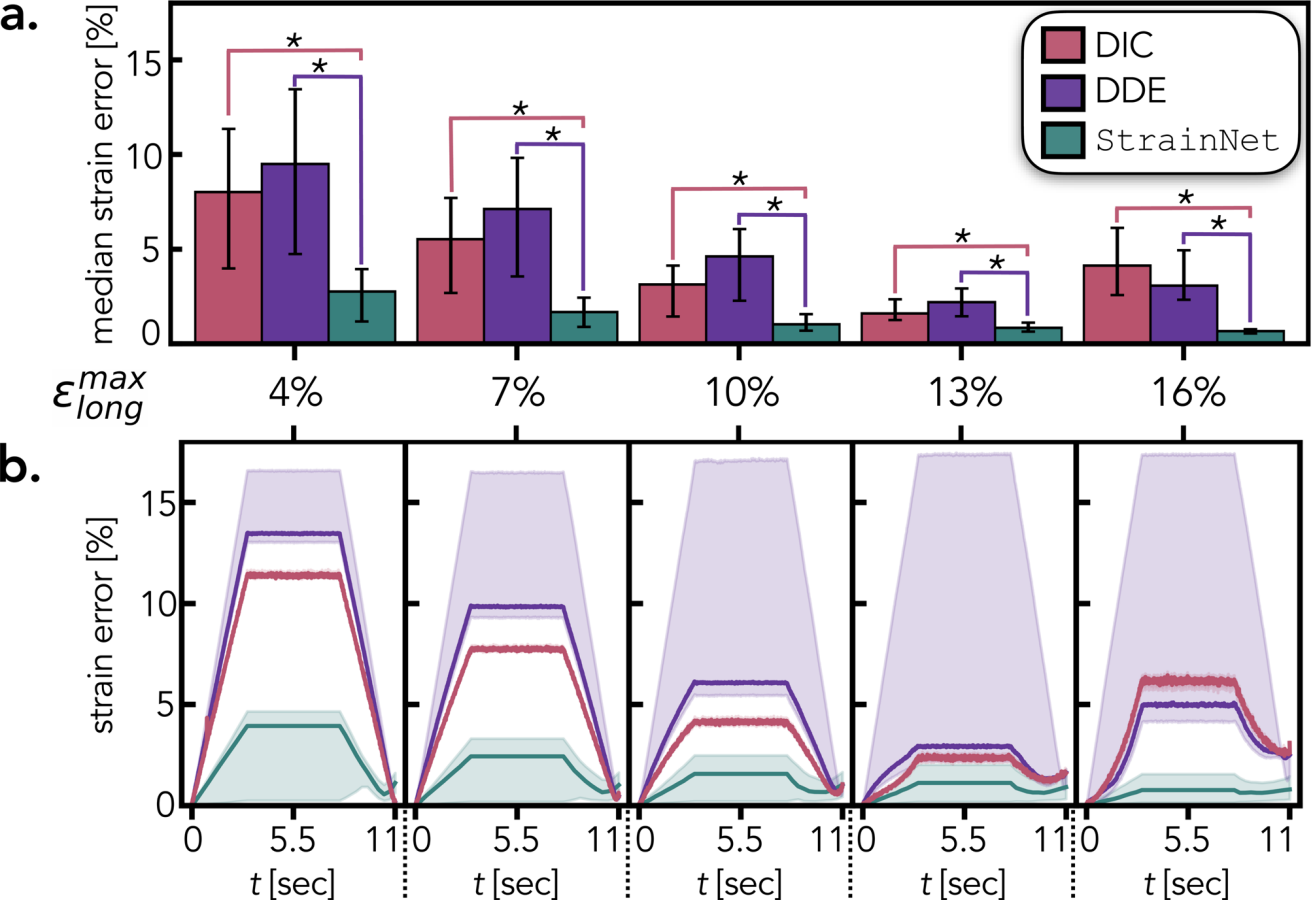
Quantitative evaluation of performance of DIC, DDE, and StrainNet on synthetic test cases. (**a**) Median strain error calculated over all ultrasound frames. Error bars indicate the first and third quartiles of the strain error. The asterisks denote a statistically significant difference between connected groups (*p* < 0.001). (**b**) Temporal strain error for each synthetic test case. The solid line indicates the median strain error for each ultrasound frame and the shadded area shows the first and third quartile range of the spatial strain error. *Errors were calculated for the region of the ultrasound image where all methods calculated strain*.

StrainNet was benchmarked against two image-based strain algorithms, digital image correlation (DIC)^36^ and direct deformation estimation (DDE)^22^, using the synthetic test cases. StrainNet significantly outperformed the traditional texture correlation algorithms in all synthetic test cases; the median strain error from StrainNet was 48-84% lower than the strain error from both DIC and DDE (Fig. 2a; $p<$ 0.001 in all strain cases). In addition to the overall performance comparison, temporal analysis of strain error further highlights the advantages of StrainNet (Fig. 2b). The accuracy of StrainNet was nearly 90% better than DIC and DDE across all test cases (solid lines in Fig. 2b). StrainNet was also 90% more precise than DDE; however, DIC was the most precise algorithm tested (filled-in area in Fig. 2b).

**Figure 3:**
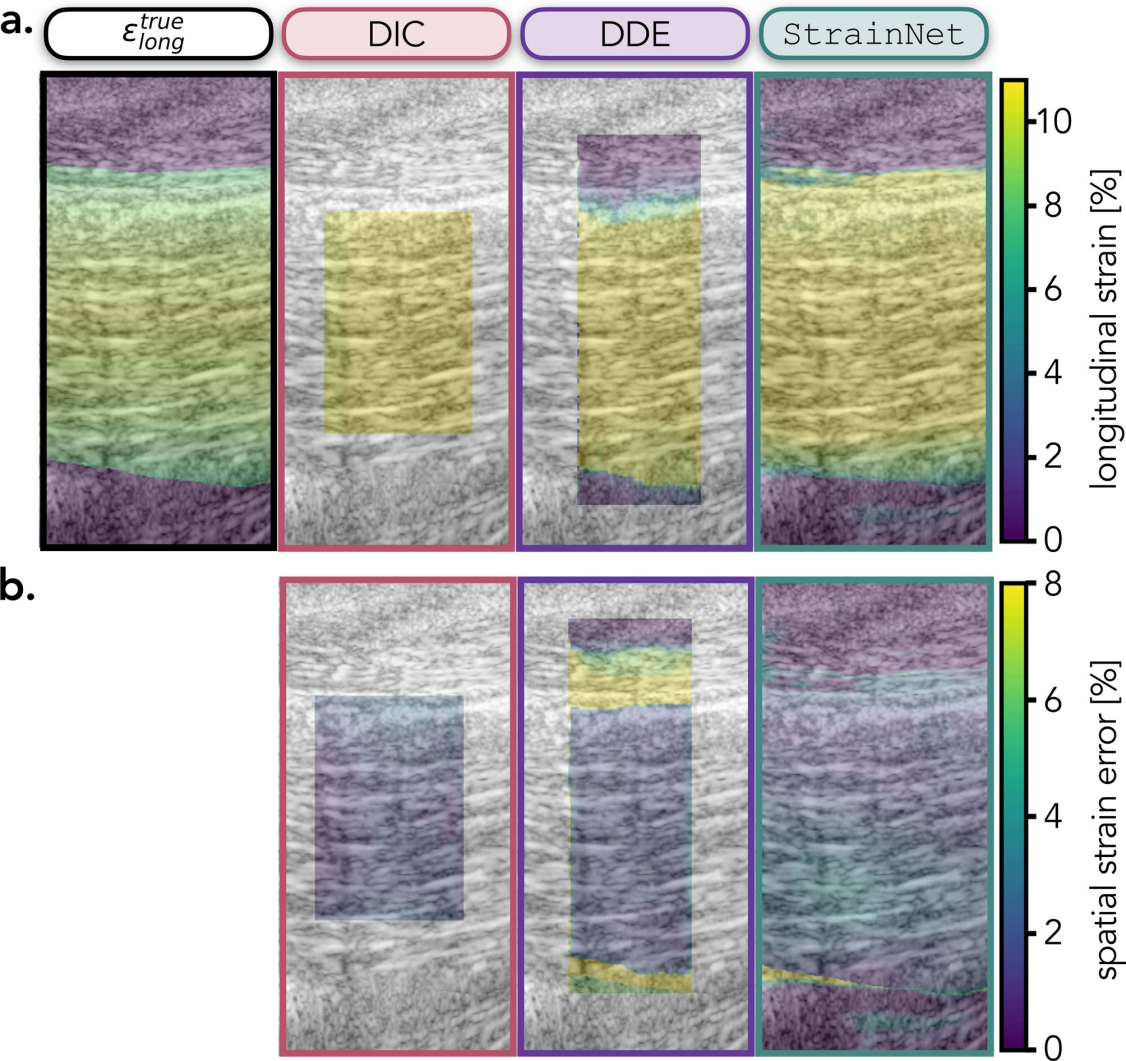
Qualitative evaluation of DIC, DDE, and StrainNet’s performance on the synthetic test case ($\epsilon _{long}^{max}$ = 10%). (**a**) From left to right: the true prescribed logitudinal strain followed by DIC-, DDE-, and StrainNet-predicted strain field during the hold period. (**b**) From left to right: spatial distribution of strain error for DIC-, DDE-, and StrainNet during the hold period.

StrainNet achieved pixel-wise strain estimation, while DIC and DDE were limited to the central area of interest (Fig. 3a). DDE and StrainNet were able to accurately capture the heterogeneity nature of the applied strain, whereas the DIC-predicted strain field was homogeneous (Fig. 3a). The DIC analysis area was limited to within the boundaries of the tendon whereas DDE and StrainNet cover both the tendon and the surrounding soft tissue, revealing large ($\sim$10%) spatial strain error at the boundary (Fig. 3b). All three methods exhibited low spatial strain error throughout the tendon during contraction and relaxation (Fig. 3b). While training StrainNet required nearly 24 h of a computational time, the inference time was less than one minute whereas DIC and DDE required approximately 10 minutes and 5 minutes, respectively, representing a 80–90% reduction in analysis time once trained.

### StrainNet enables accurate in vivo deformation estimation

**Figure 4:**
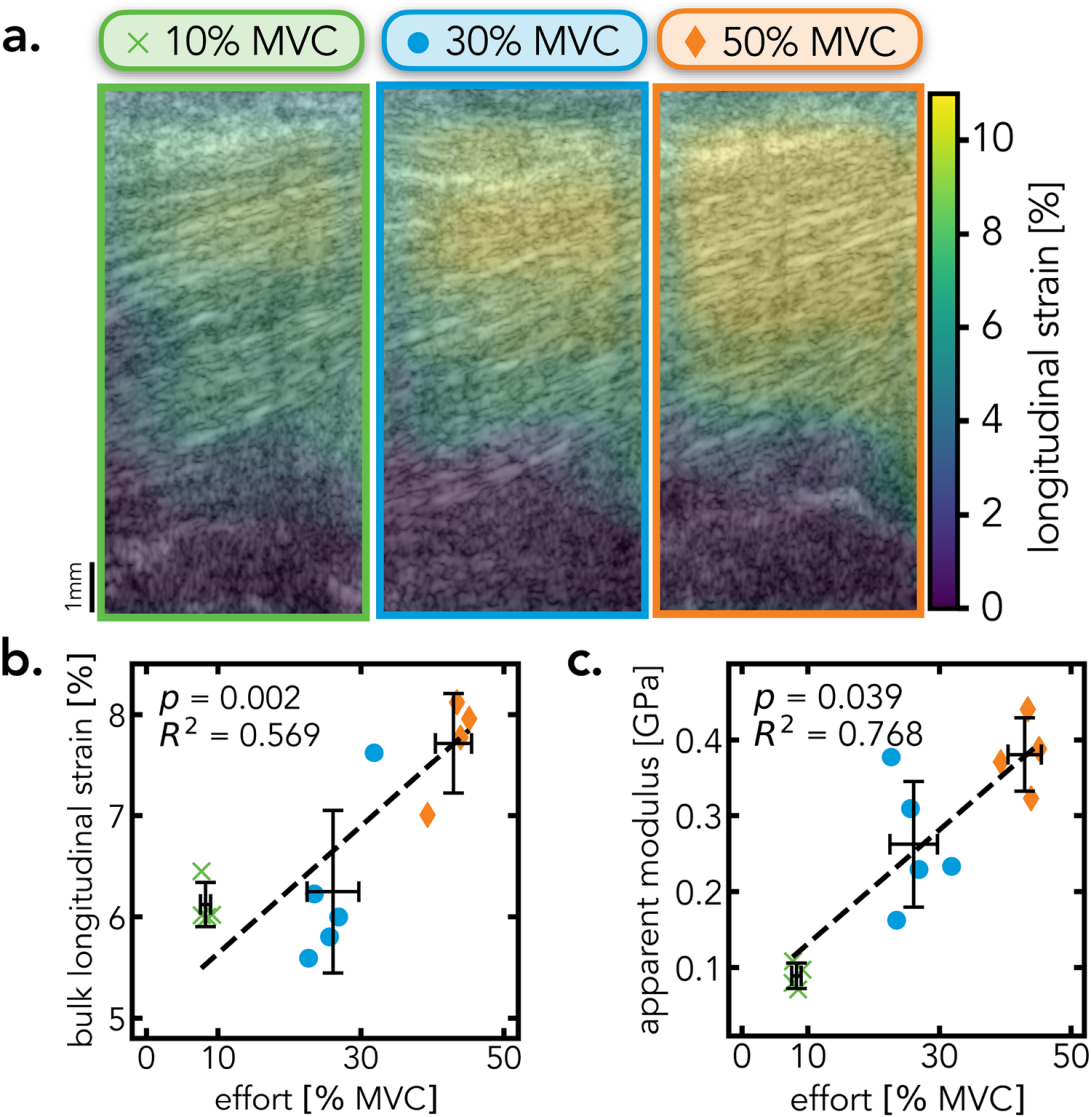
Quantitative and qualitative analysis of StrainNet applied to in vivo images. (**a**) StrainNet-measured spatial distribution of longitudinal strain throughout the tendon during the contraction to 10%, 30%, and 50% MVC. (**b**) Linear regression between the bulk longitudinal strain in the tendon and the effort exerted by the participant. (**c**) Linear regression between apparent modulus and effort level. Black cross indicates the mean ± standard deviation of the effort level and (**b**) longitudinal strain and (**c**) apparent modulus, and the dashed line is the linear regression fit.

Both DIC and DDE had difficulties tracking tissue deformations from in vivo images and many pixels were lost during analysis. StrainNet, on the other hand, was able to learn around much of the noise and predict the longitudinal strain in the tendon, which increased with the effort exerted by the participant (Fig. 4a). There was a moderate linear relationship between the StrainNet-predicted longitudinal strain and effort level (Fig. 4b; $R^2$ = 0.569, *p* = 0.002). Similarly, there was a strong linear relationship between the apparent modulus calculated with the StrainNet-predicted strain and effort level (Fig. 4c; $R^2$ = 0.768, *p*=0.039).

## Discussion

StrainNet was able to accurately measure different strain levels using ultrasound images of the flexor tendon. For synthetic datasets, StrainNet detected subtle differences in deformations with a high degree of accuracy (< 3% error), outperforming existing approaches (*e.g.*, DIC and DDE) that had median strain errors as high as 10%. Additionally, when applied to in vivo ultrasound images, StrainNet predicted a strong linear correlation between the measured strain and effort level (percentage of the MVC), further validating the performance of the model. Finally, full-field deformation predictions were able to unveil stress-strain curves, and thus measure mechanical properties within soft biological tissue under physiological boundary conditions. Taken together, these findings suggest that deep learning models have the potential to significantly advance the accuracy of in vivo biomechanics studies.

StrainNet’s novel two-stage architecture was trained on a combination of synthetic and real in vivo images^47^ to predict full-field strain maps of a sequence of images^46^. The model was able to accurately predict tissue deformation under a range of physiological strains, including those that may be encountered during daily activities. However, the strain analysis was limited to a single imaging plane with the aid of a custom mount to minimize off-plane motion. Clinical settings often perform ultrasound imaging freehand, potentially introducing off-plane motion. Understanding the impact of off-plane motion on model performance or transitioning to a 3D imaging environment may make StrainNet more robust to clinical settings. Additionally, while StrainNet outperformed traditional image texture algorithms in this study, advances in image-based strain measurement have steadily grown^51^, and it would be beneficial to continue benchmarking them against deep learning models.

There are several limitations to our model that will be addressed in future work. First, the model was evaluated on a single tissue type on a single participant. Expanding its application to a wider range of tissue types across a larger cohort is a key next step. Additionally, the model is currently to handle only three types of bulk deformation and the model was trained on a generalized mathematical model of tendon mechanics (Section S3 in the Supplemental Information), which is not representative of all biological tissues. The training set would have to be expanded and further generalized to be aligned to other biological tissues under different imaging conditions and experimental configurations. As such, a tutorial on how to customize the training set and its mechanical model (e.g., hard-tissue CT, soft-tissue MRI, or a combination of both) was included. Finally, StrainNet was trained with a loss function that did not consider the feasibility of the predicted strain field. Future work could also incorporate the underlying physics (e.g., conservative equations) into the training process leading to more physically accurate strain fields.

The potential applications of StrainNet are vast and promising. StrainNet significantly surpassed traditional image texture correlation methods in controlled environments, such as synthetic test cases (Fig. 2). Moreover, in more complex settings where image texture correlation is susceptible to errors caused by image artifacts, StrainNet consistently delivered accurate and expected tissue deformation levels (Fig. 4), in line with previous reports^37–39^. Furthermore, the measured tissue mechanical properties aligned with those previously reported for human patellar and Achilles tendons with similar experimental procedures in vivo (Section S6 in the Supplemental Information)^37,52–55^. Taken together, our results suggest that StrainNet may be aligned to a broad array of biomedical applications, such as in vivo imaging studies of muscle function, blood flow, and tissue viability. In summary, the design and capabilities of StrainNet hold immense potential for quantifying biomechanical metrics, leading to substantial progress in assessing soft tissue deformation.

## Methods

### StrainNet architecture and training

**Figure 5:**
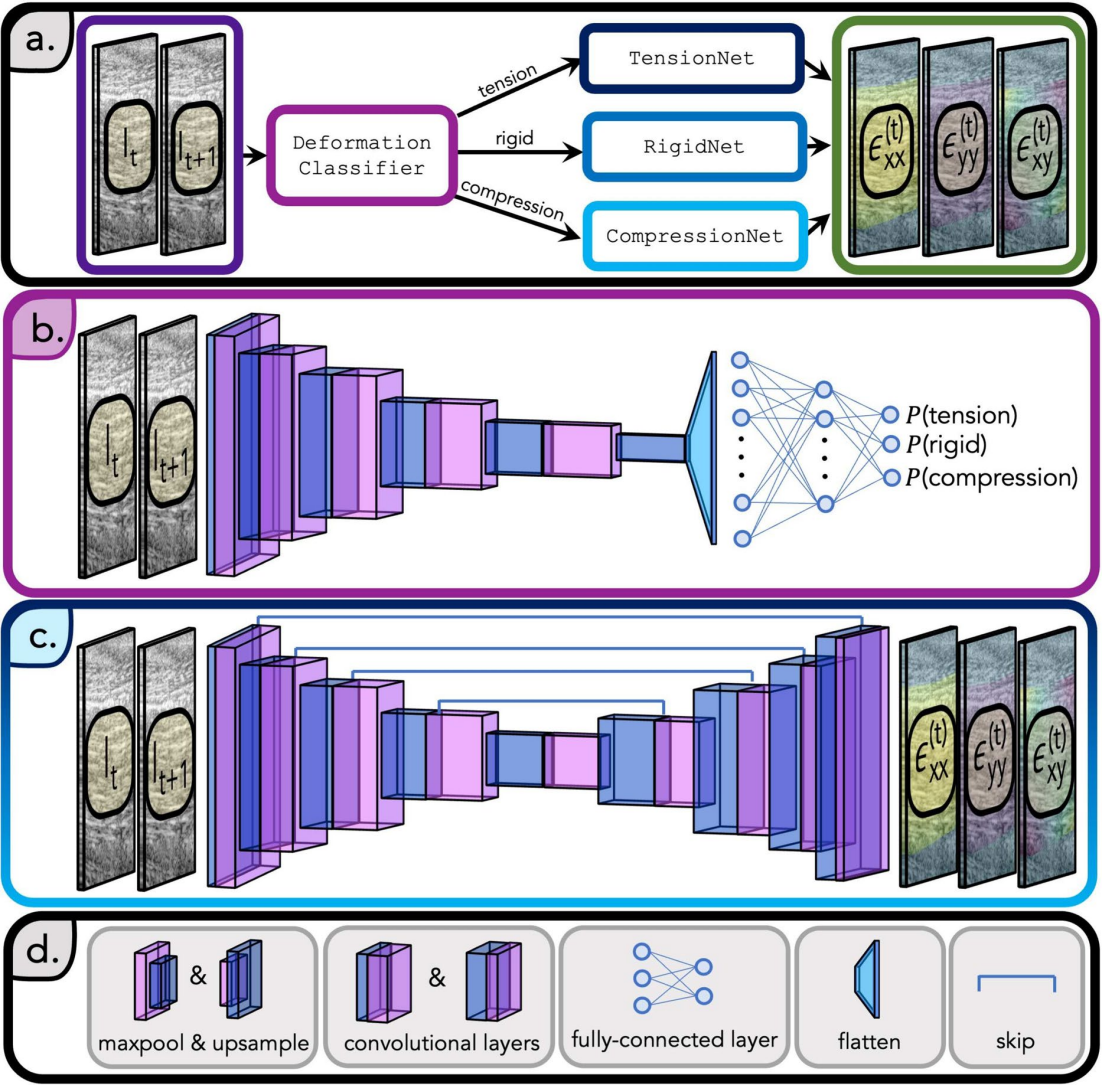
Architecture of StrainNet. (**a**) StrainNet includes a deep neural network trained to predict a relationship between two ultrasound frames, $I_t$ and $I_{t+1}$ and tendon strain at frame *t*, $\epsilon _{xx}^{(t)}$, $\epsilon _{xy}^{(t)}$, $\epsilon _{yy}^{(t)}$. StrainNet comprises two stages, where the first stage was the DeformationClassifier and the second stage included TensionNet, CompressionNet, and RigidNet. The input to the first stage is a pair of ultrasound images, the output is its class deformation type (tension, compression, or rigid). The input to the second stage is again a pair of ultrasound images as well as its deformation type, and the output is the full strain field of the tendon. (**b**) DeformationClassifier is composed of convolutional layers, max pooling, and Rectified Linear Unit (ReLU) activation functions. The resulting features from the input image pair are flattened and passed through a fully-connected neural network to predict the probability of the image pair undergoing tension, compression, or rigid body motion. (**c**) The architecture of TensionNet, CompressionNet, and RigidNet included convolutional layers, max pooling, upsampling, skip layers, and ReLU activation functions. The input image pair is passed through the corresponding network to predict the full strain field ($\epsilon _{xx}^{(t)}$, $\epsilon _{xy}^{(t)}$, $\epsilon _{yy}^{(t)}$). (**d**) Blocks in (**b**) and (**c**) were connected by ReLU activation functions and utilized batch normalization.

The StrainNet architecture was specifically designed to handle the unique challenges present with in vivo image analysis. Specifically, StrainNet was developed and trained to predict strain within high-frequency ultrasound images of FDS tendons undergoing contraction, as described above (“Human flexor tendons undergoing contraction” section). For each contraction-relaxation cycle, a set of ultrasound images $(I_{t})_{t=1}^T$ are collected, where *T* represents the total number of frames during the cycle. Therefore, the goal of StrainNet was to predict the strain field $\epsilon _{xx}^{(t)}$, $\epsilon _{xy}^{(t)}$, and $\epsilon _{yy}^{(t)}$ for each frame or time point *t*. To achieve this, StrainNet was designed as a two-stage deep neural network architecture (Fig. 5a).

The architecture was constructed to first classify the image pair as undergoing tension, compression, or rigid body motion, and then to apply an appropriate neural network to predict the strain field within the tendon (Fig. 5a).

The first stage of the architecture, the DeformationClassifier, was a CNN that classifies the type of deformation (e.g., tension, compression, or rigid motion) within each ultrasound image pair $I_{t}$ and $I_{t+1}$ (Fig. 5a). The cumulative strain is relative to the first image. The DeformationClassifier consisted of a series of convolutional layers, max-pooling layers, and fully connected layers. The convolutional layers extracted features from the image, while the max-pooling layers reduced dimensionality. The fully connected layers were used to make the final classification (Fig. 5b).

Once the type of deformation between $I_{t}$ and $I_{t+1}$ was classified, the *same* image pair was passed to one of three neural networks: TensionNet, CompressionNet, or RigidNet (Fig. 5a). These networks predict the strain field from the input image pair and were based on the UNet architecture, a popular biomedical image segmentation deep-learning architecture^56^. The UNet architecture has an encoder-decoder structure, with the encoder extracting features and the decoder up-sampling feature maps to the original image size. The encoder and decoder in TensionNet, CompressionNet, and RigidNet were composed of convolutional layers, max-pooling layers, up-sampling layers, and Rectified Linear Unit (ReLU) activation functions (Fig. 5c,d). Skip connections between the encoder and decoder were included to help improve strain field prediction quality by reducing vanishing gradients^57^.

Each pair of images in the ultrasound image sequence is passed through both stages, allowing StrainNet to predict the full strain field at each time point except *T*. As a result, StrainNet is able to predict the longitudinal, transverse, and shear strain fields across the entire tendon region, providing a full-field strain map, $(\epsilon _{xx}^{(t)}$, $\epsilon _{xy}^{(t)}$, $\epsilon _{yy}^{(t)})_{t=1}^{T-1}$.

To effectively train StrainNet, a diverse training set was created with image pairs of deformation fields that emulated real-world observations and image artifacts commonly encountered in medical imaging (e.g., random noise). The training set included 1,250 experimental image pairs from a different participant than the one used for the in vivo experiment and 3750 synthetically generated image pairs. Training set generation involved utilizing a generalized mathematical model of tendon mechanics, prescribing the non-linear strain fields onto collected ultrasound images of the tendon, and adding noise to simulate real-world imaging conditions. Deformation and noise parameters were randomly sampled from uniform probability distributions, ensuring a robust dataset for learning the strain measurement task. The detailed process of generating the training set, including the acquisition of experimental data, image preprocessing, and the combination of synthetic and experimental examples, can be found in Section S4 in the Supplemental Information.

Following the creation of the training set, the StrainNet model was trained using a combination of loss functions tailored to the specific tasks of each subnetwork. For the DeformationClassifier, a cross-entropy loss function was utilized and defined as

1 $$\begin{aligned} \mathcal {L}_{\text {CE}}(p, y) = -\sum _{i=1}^{C} y_i \log (p_i), \end{aligned}$$

where *p* represents the predicted class probabilities, *y* is the true one-hot encoded class label, and *C* is the number of classes (tension, rigid, and compression).

For the other three models, TensionNet, CompressionNet, and RigidNet, the mean $\ell _2$ loss function was used and expressed as

2 $$\begin{aligned} \mathcal {L}_{\ell _2}(\epsilon ^{pred}, \epsilon ^{true}) = \frac{1}{N} \frac{1}{P} \sum _{n=1}^{N} \sum _{p=1}^{P} \sum _{i=1}^{2} \sum _{j=1}^{2} \left| \widehat{\epsilon }_{ij, p}^{(n)} - \epsilon _{ij, p}^{{(n)}} \right| ^2, \end{aligned}$$

where $\widehat{\epsilon }^{(n)}_{ij,p}$ and $\epsilon ^{(n)}_{ij,p}$ denote the *ij*-th component of the true and predicted (i.e., longitudinal, transverse, and shear) at pixel *p* of the *n*-th example in the training set, respectively. Here, we are summing over all of the pixels in the image *P* and over the number of examples in the training set *N*.

The training process began by splitting the training set into 80% training and 20% validation sets. Training was conducted for 100 epochs using the Adam optimizer (PyTorch^58^ 1.12.1) on a NVIDIA K100 16GB graphics processing unit (GPU) with a learning rate of 0.001. Different batch sizes were employed for the sub-models to accommodate their specific training requirements. For the DeformationClassifier, a batch size of 100 was used to take advantage of parallel processing and to reduce the noise in gradient updates. In contrast, a smaller batch size of 10 was utilized for the TensionNet, CompressionNet, and RigidNet models, allowing for more frequent weight updates and improved convergence properties. Training of the DeformationClassifier required 4 h, whereas TensionNet, CompressionNet, and RigidNet needed approximately 8 h to train. The combination of these hyperparameters, the GPU, and the optimizer facilitated successful training of StrainNet, enabling it to learn the relationships between ultrasound images of tendons and their corresponding strain fields. The trained model reached loss (Eq. (2)) of 2.4% with a 100% classification accuracy from the DeformationClassifier on the validation set, demonstrating the model’s ability to accurately predict the strain field from ultrasound images.

### Strain analysis method validation

StrainNet’s performance and accuracy was compared to two existing texture correlation algorithms, including DIC^36^ and DDE^22^. All three techniques were applied to all synthetic test cases. Hyperparameters of DIC and DDE such as subset size and step size were tuned to maximize accuracy of the strain field prediction, while the shape function was linear for both methods. Given the applied strain tensor, the spatial strain error was calculated as

3 $$\begin{aligned} \text {spatial strain error} = \sqrt{ \sum _{i=1}^2 \sum _{j=1}^2 \left( \widehat{\epsilon }_{ij,p}^{(t)} - \epsilon _{ij,p}^{(t)} \right) ^2 } \end{aligned}$$

where $\widehat{\epsilon }_{ij,p}^{(t)}$ and $\epsilon _{ij,p}^{(t)}$ represent the true and predicted strain tensor at pixel *p* and time *t*, respectively. To robustly evaluate the performance, strain error and median strain error were calculated as

4 $$\begin{aligned} \text {strain error}&= \underset{p_{\text {overlap}}}{{\text {median}}} \hspace{0.2cm} \text {spatial strain error}\end{aligned}$$

5 $$\begin{aligned} \text {median strain error}&= \underset{p_{\text {overlap}}, t}{{\text {median}}} \hspace{0.2cm} \text {spatial strain error} \end{aligned}$$

where $p_{\text {overlap}}$ denotes the median strain ever taken over the shared region of interest for all three methods and subscript *t* denotes the median over time. Therefore, the strain error in (4) represents the error *across the image* the median strain error in (5) represents the error across the images *throughout the full contraction-relaxation cycle*.

To compare the strain error for each synthetic test case between StrainNet and DIC, as well as between StrainNet and DDE, permutation tests of the strain errors were conducted for each test case.

### Experimental strain analysis and mechanical property estimation

StrainNet, DIC, and DDE were then applied to the experimental images. To quantify the bulk tendon mechanical behaviour, the bulk longitudinal strain during the hold period was calculated as the median longitudinal strain over the tendon region

6 $$\begin{aligned} \text {bulk longitudinal strain} = \underset{p_\text {tendon}, t_{hold}}{{\text {median}}} \hspace{0.2cm} \epsilon _{long}^{(t)} \end{aligned}$$

where $p_\text {tendon}$ and $t_{hold}$ represent the region of the image containing the tendon and the time over the hold period, respectively. Repeated measures linear regression was performed to examine the relationship between the effort level and the corresponding bulk longitudinal strain for each of the three methods (StrainNet, DIC, and DDE).

The longitudinal strains were then used to estimate tendon mechanics. First, the longitudinal force in the tendon was calculated by related the grip force to the force in the FDS tendon^59^. Longitudinal stress was calculated by dividing the tendon force by its cross-sectional area, which was manually segmented from ultrasound images at the beginning of each trial (i.e., in the ’undeformed’ state). Subsequently, the tendon’s apparent modulus was calculated as the slope of the linear region of each trial’s stress-strain curve (Supplementary Fig. S5b). A full description of the modulus estimation can be found in Section S7 in the Supplemental Information. Finally, repeated measures linear regression was performed to examine the relationship between the effort level and the corresponding bulk longitudinal strain for each of the three methods (StrainNet, DIC, and DDE).

The significance level for all permutation and linear regression tests was set to 0.05.

### Supplementary Information

Supplementary Information.

## Data Availability

The pre-trained models are available on the project page, strainnet.github.io, as well as a detailed tutorial for implementing StrainNet in any desired experimental setup with any biological tissue. The code is publicly available at github.com/reecehuff/StrainNet. All data and code questions and requests should be addressed to R.D.H. at rdhuff@berkeley.edu.
